# Sense of coherence and its context with demographics, psychological aspects, lifestyle, complementary and alternative medicine and lay aetiology

**DOI:** 10.1007/s00432-023-04760-9

**Published:** 2023-04-20

**Authors:** B. Bargehr, L. Fischer von Weikersthal, C. Junghans, B. Zomorodbakhsch, C. Stoll, F.-J. Prott, S. Fuxius, O. Micke, J. Hübner, J. Büntzel, C. Hoppe

**Affiliations:** 1 Klinik für Innere Medizin II, Hämatologie und Internistische Onkologie, Universitätsklinikum Jena, Am Klinikum 1, 07747 Jena, Germany; 2 Gesundheitszentrum St. Marien GmbH, Praxis für Hämatologie und Internistische Onkologie, Mariahilfbergweg 7, 92224 Amberg, Germany; 3 Paracelsus Klinik am Schillergarten Bad Elster, Martin-Andersen-Nexö-Straße 10, 08645 Bad Elster, Germany; 4 üBAG/MVZ Onkologische Kooperation Harz GbR, Kösliner Straße 14, 38642 Goslar, Germany; 5 Klinik Herzoghöhe Bayreuth, Kulmbacher Straße 103, 95445 Bayreuth, Germany; 6 Strahlentherapie am St. Josef Krankenhaus, Beethovenstraße 20, 65189 Wiesbaden, Germany; 7 Onkologische Schwerpunktpraxis Heidelberg, Kurfürsten-Anlage 34, 69115 Heidelberg, Germany; 8 Franziskus Hospital, Klinik für Strahlentherapie und Radioonkologie, Kiskerstraße 26, 33615 Bielefeld, Germany; 9 Klinik für HNO-Erkrankungen, Kopf-Hals-Chirurgie, Interdisziplinäre Palliativstation, Südharz Klinikum Nordhausen, Dr.-Robert-Koch-Straße 39, 99734 Nordhausen, Germany

**Keywords:** Sense of coherence, Cancer, General life satisfaction, Resilience, Spirituality, Self-efficacy

## Abstract

**Purpose:**

For patients with a cancer diagnosis, coping abilities are of high importance. Cancer patients with a high sense of coherence may cope better. The purpose of this study is to learn more about the correlation of sense of coherence and different aspects, such as demographics, psychological factors, lifestyle, complementary and alternative medicine (CAM) and lay aetiology.

**Methods:**

A prospective cross-sectional study was performed in ten cancer centres in Germany. The questionnaire consisted of ten sub-items, collecting information about sense of coherence, demographics, general life satisfaction, resilience, spirituality, self-efficacy, physical activity and sports, nutrition, CAM methods and cancer causes.

**Results:**

As many as 349 participants were evaluable. The mean sense of coherence score was *M* = 47.30. Significant associations were shown for sense of coherence and financial situation (*r* = 0.230, *p* < 0.001), level of education (*r* = 0.187, *p* < 0.001), marital status (*η* = 0.177, *p* = 0.026) and time interval since diagnosis (*r* = − 0.109, *p* = 0.045). Sense of coherence and resilience correlated on a high level, as well as spirituality, self-efficacy and general life satisfaction (*r* = 0.563, *r* = 0.432, *r* = 0.461, *r* = 0.306, *p*’s < 0.001).

**Conclusion:**

Several aspects, such as demographics and psychological factors, have a great influence on the sense of coherence. To help patients to cope better, physicians should try to strengthen sense of coherence, resilience and self-efficacy and, at the same time, consider patients’ individual background such as level of education, financial capacity and emotional support by family members.

## Introduction

Every year, thousands of patients are faced with a newly discovered cancer diagnosis. In 2020, the International Agency for Research on Cancer (IARC) published the GLOBOCAN statistics, estimating that 19 million new cancer diagnoses were diagnosed worldwide, while at the same time a total of 10 million patients died from their cancer (Sung et al. 2021). The diagnosis often has a strong impact on social life and psychological wellbeing. Despair, hopelessness and fear are just a few of the emotions patients go through in this context. While some patients linger in the spiral of hopelessness and lapse into listlessness, other patients show an urge to actively participate in therapy (Ciarlo et al. 2021). Coining concepts are the sense of coherence and self-efficacy.

Antonovsky first coined the term “sense of coherence” in 1979 in his model of salutogenesis in his book *Health, Stress and Coping*. Based on this, he defined dealing or coping with stressors on the basis of one's own abilities. As described by Konaszewski et al. people with a higher sense of coherence can cope better with situations and avoid stressors (2021). Thus, the sense of coherence is composed of three terms: meaningfulness, comprehensibility and manageability.

In contrast, self-efficacy was first defined by Bandura in 1977 as a person’s belief that he or she can influence situations, such as health, through his or her own actions. Konaszewski et al. described a positive correlation between high self-efficacy and a problem-oriented style of coping with stress (2021).

Resilience is another important factor associated to the trauma related to a cancer diagnosis and describes the totality of acquired resistance abilities that a patient has developed against negative influences and stressors in the course of life, so that, under certain circumstances, there are no psychological and/or physical limitations (Bonanno et al. 2011). In this regard, important factors such as education, psychological integrity, spirituality and faith/hope may play an essential role (Duggal et al. 2016; Huey et al. 2020; Manomenidis et al. 2019).

Complementary and alternative medicine (CAM) comprises methods which allow the patient to actively participate in therapy. The different methods in CAM can be individually adapted to one’s own needs and life circumstances. A large number of patients use CAM to strengthen the body’s own forces, but also to strengthen their own immune system or to detoxify the body (Ciarlo et al. 2021; Huebner et al. 2014).

Factors influencing sense of coherence in Japanese high school students have been described by Omiya et al. and include factors such as family relationships, high school life and autism spectrum tendency (Omiya et al. 2020).

The main goal of this study is to analyse the coherence of patients with cancer in more detail, to identify promoting factors and to enlighten the meaningfulness of aspects that are associated with the sense of coherence. Furthermore, the correlation between coherence and demographics, psychological factors, lifestyle, CAM use and lay aetiology with respect to cancer will be examined.

## Methods

### Study design and participants

Between March and July 2021, a prospective cross-sectional study was performed in ten cancer centres in Germany (six oncological departments of hospitals, one rehabilitation clinic and three oncological offices), where a printed questionnaire was handed out to outpatient cancer patients. The patients’ consent was given by completing the questionnaire. The data were processed anonymously. To actively take part in this study, participants firstly had to be diagnosed with cancer, and secondly had to be undergoing current cancer treatment. Patients needed to be able to understand the German language to answer all questions, and as well needed to be over 18 years old. Furthermore, participants had to have a valid sense of coherence score to be included into this study.

### Questionnaire

The questionnaire consisted of five parts. The first part queried demographic information such as age, sex, financial status, marital status, religion affiliation, educational background, type of cancer and time interval since cancer diagnosis.

The second part contained questions about five different psychological aspects:

1. The L-1 short scale (Lebenszufriedenheit-1 Kurzskala; Life satisfaction short scale) is a single-item-scale on a 10-point Likert scale to assess general life satisfaction reaching from “not satisfied at all” (0) to “fully satisfied” (10) with a re-test-reliability of 0.67 after 6 weeks (Beierlein et al. 2014).
2. The RS-13 scale (Leppert et al. 2008) is a short version of the RS-25 scale (Schumacher et al. 2005) to measure patients’ resilience. It consists of 13 items on a 7-point Likert scale (1 = “I don’t agree at all” to 7 = “I fully agree”) with the two factors “Acceptance of Self and Life” and “Personal Competence” added by the authors, Wagnild and Young, in 1993 (Leppert et al. 2008). The re-test reliability of the RS-13 scale is 0.61 and the internal consistency is excellent (Cronbach’s *α* = 0.9). The resilience score can be divided into “low” (13–66 points), “moderate” (67–72 points) and “high” (73–91 points). The mean value of the given answers was used if one item was missing. Questionnaires with more than one answer missing were excluded.
3. The SOC-L9 questionnaire is a short version from Leipzig (Schumacher et al. 2000) to measure the sense of coherence with nine items on a 7-point Likert scale with changing answers relating to the three subcategories “comprehensibility”, “manageability” and “meaningfulness”. The score range is from 9 to 63 points. The internal consistency is very good (Cronbach’s *α* = 0.87). In case of a missing item, the same procedure as for the RS-13 scale was carried out.
4. The FACIT-Sp12 questionnaire (Functional Assessment of Chronic Illness Therapy-Spiritual Well-Being Scale) with 12 items on a 5-point Likert-scale (0 = “not at all” to 4 = “very much”) evaluates the spirituality in cancer patients and consists of three subscales “meaning”, “peace” and “faith”. The score range is from 0 to 48 points. The internal consistency of the subscales was good (Cronbach’s *α* = 0.81–0.88) (Bredle et al. 2011).
5. The ASKU questionnaire (Allgemeine Selbstwirksamkeit Kurzskala; Short Scale for Measuring General Self-efficacy Beliefs) captures information about self-efficacy with three items on a 5-point Likert scale (1 = “doesn’t apply at all” to 5 = “fully applies”) (Beierlein et al. 2013). The short scale’s reliability is between 0.81 and 0.86. If one item is missing, the mean value “3” was taken to complete the scale.

The third part was determining two lifestyle aspects:

1. The daily physical activity before getting diagnosed with cancer and the current daily physical activity was scanned with a short questionnaire giving each section four possible time specifications such as “less than 10 min”, “11–30 min”, “31–60 min” and “more than 60 min”. To investigate participants’ daily sports before their cancer diagnosis and their current daily sports, there was a choice of three possible answers “0–2 h”, “2–4 h” and “more than 4 h” (Höh et al. 2018). Answer options refer to up-to-date recommendations from the German S3-guideline “Complementary medicine in the treatment of cancer patients” (Leitlinienprogramm Onkologie 2021).
2. To asses nutrition habits, a short version of the AFHC questionnaire (The Adolescent Food Habits Checklist) consisting of 12 items (instead of 23 items) was used (Johnson et al. 2002). For every healthy response 1 point can be scored. Questions deal with eating habits and the consumption of sugar, fat, meat, fruits, vegetables and soft drinks.

The fourth part collected information about CAM methods over a 10-item shortened questionnaire originally developed by the Working Group Prevention and Integrative Oncology of the German Cancer Society (PRIO) (Huebner et al. 2014). The questionnaire was divided into two sections asking patients whether they have ever used and whether they were currently using any CAM methods, such as vitamin D, vitamin C, selenium, zinc, curcumin, mistletoe, and vitamin B17/apricot kernels, which are summarised as biological CAM methods, and acupuncture, Chinese herbs/tea, and homeopathy, which are summarised as holistic CAM methods.

In the fifth and last part, patients could choose from the given 28 reasons as many as they thought to be the cause of their cancer disease. If none of them applied, participants could also state their own explanations. Josfeld et al. evaluated self-efficacy and its correlation with the use of CAM, lifestyle and lay aetiology, whereas Welter et al. examined lay aetiology, self-efficacy and patient activation among cancer patients (2022, 2021).

### Statistical analyses

Statistical analyses were conducted using Statistical Package for Social Science (IBM SPSS), version 28. Eta’s, Spearman’s and Pearson’s correlation coefficients were calculated to express a possible correlation between sense of coherence and demographics, psychological aspects, lifestyle, complementary and alternative medicine and lay aetiology. To assess group differences, ANOVA with Post-Hoc-test (Bonferroni) for more than two groups, Mann–Whitney-U-test/independent sample t-test for only two groups and dependent sample t-test for before/after group comparisons were carried out. A *p* < 0.05 was regarded as significant.

## Results

### Demographics

Altogether, 451 patients from 10 outpatient cancer departments in Germany filled out the questionnaire. One hundred and two participants were excluded in this study as they did not have a valid sense of coherence score. Thus, 349 patients took place in further analyses.

From all participants, 222 (63.6%) were female and 118 (33.8%) were male, nine (2.6%) of them did not state any gender. The mean age of the participators was 61.80 years with a range from 33 to 85 years.

Additional information about the total imposed demographic data is shown in Table 1.

**Table 1:** Demographic data (*N* = 349)

Data	*n*	%
Financial managing without income		
1–6 months	121	34.7
6 months–1 year	80	22.9
More than 1 year	131	37.5
No answer	17	4.9
Marital status		
Single	21	6
Married	26	7.4
Relationship	246	70.5
Divorced	33	9.5
Widowed	23	6.6
Religion		
Christian	189	54.2
Muslim	1	0.3
Other	2	0.6
None	152	43.6
No answer	5	1.4
Level of education		
No degree	8	2.3
Middle school (8th/10th grade)	228	65.3
A-levels/high school graduation	23	6.6
University/college degree	86	24.6
No answer	4	1.1
Type of cancer		
Breast	118	33.8
Head and neck	42	12
Colorectal	47	13.5
Gastrointestinal tract	27	7.7
Leukaemia, Lymphoma	25	7.2
Prostate	12	3.4
Lung	15	4.3
Pelvis	16	4.6
Urogenital tract	6	1.7
Others	23	6.6
No answer	18	5.2
Time interval since diagnosis		
Less than 1 year	153	43.8
1–3 years	103	29.5
3–6 years	40	11.5
More than 6 years	40	11.5
No answer	13	3.7

### Sense of coherence

The mean value of the sense of coherence score was 47.30 with a score range from 9 to 63 possible points. Outcomes of the correlation analyses are stated in Table 2.

**Table 2:** Correlations of sense of coherence and reported variables

Variable	*n*	Correlation coefficient	*p*
Age	346	r = 0.006	0.916
Gender	340	η = 0.026	0.638
*Financial managing without income*	*332*	*r *= *0.230*	*< 0.001*
*Marital status*	*349*	*η* = *0.177*	*0.026*
Religion	344	η = 0.062	0.723
*Level of education*	*345*	*r* = *0.187*	*< 0.001*
Type of cancer	331	η = 0.105	0.936
*Time interval to diagnosis*	*336*	*r* = –*0.109*	*0.045*
*General life satisfaction*	*320*	*r* = *0.306*	*< 0.001*
*Resilience*	*341*	*r* = *0.563*	*< 0.001*
*Spirituality*	*339*	*r* = *0.432*	*< 0.001*
*Self-efficacy*	*341*	*r* = *0.461*	*< 0.001*
Daily activity before cancer diagnosis	312	r = 0.102	0.072
*Current daily activity*	*320*	*r* = *0.118*	*0.035*
Daily sports before cancer diagnosis	299	r = 0.016	0.778
Current daily sports	290	r = – 0.029	0.617
Nutrition habits	341	r = 0.102	0.061
Biological CAM usage ever	296	r = – 0.040	0.492
Holistic CAM usage ever	269	r = 0.038	0.538
Current biological CAM usage	276	r = – 0.069	0.25
*Current mistletoe usage*	*200*	*r* = − *0.200*	*0.005*
*Environmental pollutants*	*349*	*r* = − *0.116*	*0.031*
*Lack of sleep*	*349*	*r* = − *0.212*	* < 0.001*
*Working conditions*	*349*	*r* = − *0.297*	*< 0.001*
*Stress*	*349*	*r* = − *0.280*	*< 0.001*
*Unhealthy nutrition*	*349*	*r* = − *0.153*	*0.004*
*Overweight*	*349*	*r* = − *0.164*	*0.002*
*Little physical activity*	*349*	*r* = − *0.164*	*0.002*
*Death of a close relative*	*349*	*r* = − *0.126*	*0.019*
*Depression or other mental diseases*	*349*	*r* = − *0.293*	*< 0.001*
*Medicine*	*349*	*r* = − *0.109*	*0.043*
*No such thing*	*349*	*r* = *0.127*	*0.017*

The values in italics are significant results

### Sense of coherence and demographic data

A higher sense of coherence went along with a better income (*r* = 0.203, *p* < 0.001) and a better level of education (*r* = 0.187, *p* < 0.001). Group differences in financial managing without income showed a significant result: the group that could manage more than one year without income had a higher sense of coherence compared to the group that could manage only 1–6 months without income (*p* < 0.001, *M*_*Diff*_ = 4.427, *95% CI*[1.93, 6.92]). Moreover, group differences in the level of education showed a significant outcome: the group with a university/college degree showed a higher sense of coherence compared to the group with a middle school degree (*p* = 0.010, *M*_*Diff*_ = 3.362, *95% CI*[0.56, 6.17]).

In addition, the marital status weakly correlated with the sense of coherence (*η* = 0.062, *p* = 0.026). Group differences in marital status demonstrated a significant result: the group of patients who lived in a relationship revealed a greater sense of coherence compared to the group of divorced patients (*p* = 0.010, *M*_*Diff*_ = 5.147, *95% CI*[0.75, 9.54]).

Participants who had been diagnosed with cancer a long time ago had a significant lower sense of coherence (*r* =  − 0.109, *p* = 0.045). Furthermore, no significant correlations between sense of coherence and age, gender, religion and type of cancer could be detected (Table 2).

### Sense of coherence and psychological factors

General life satisfaction, resilience, spirituality and self-efficacy were among psychological aspects in this study. The mean value of general life satisfaction was 6.34 and strongly correlated with the sense of coherence (*r* = 0.306, *p* < 0.001).

A mean resilience score of 69.15, which is located in the moderate range of the scale, was achieved; 129 participants had a low score (37.0%), 59 patients got a moderate score (16.9%), 153 attendants gained a high score (43.8%) and eight patients (2.3%) did not have a valid resilience score. The correlation between sense of coherence and resilience was significant on a high level (*r* = 0.563, *p* < 0.001).

The mean spirituality score was 31.57 and the correlation with sense of coherence showed that patients who identified themselves as spiritual had a better sense of coherence (*r* = 0.432, *p* < 0.001).

Moreover, the mean value of self-efficacy was 3.91 out of five possible points and it could also be demonstrated that the sense of coherence correlated with self-efficacy on a high level (*r* = 0.461, *p* < 0.001).

### Sense of coherence and lifestyle

Lifestyle in this study summarised the factors daily activity and sports before getting diagnosed with cancer and the current daily activity and sports, and as well the participants’ food habits. Results showed that the majority of cancer patients were physically active at an average of more than 60 min every day before getting diagnosed and of at least 31–60 min every day at present. The current daily activity was positively correlated to their sense of coherence score (*r* = 0.118, *p* = 0.035), whereas no significant correlation between daily activity before getting diagnosed with cancer and sense of coherence could be detected (Table 2). Group differences in daily activity before getting a cancer diagnosis stated a significant result: the group in which patients were more than 60 min active every day before getting diagnosed had a higher sense of coherence compared to the group which was only active between 31 to 60 min every day before getting diagnosed (*p* = 0.005, *M*_*Diff*_ = 4.395, *95% CI*[0.95, 7.84]). In addition, group differences in current daily activity every day showed a significant outcome: the group currently more than 60 min active every day revealed a higher sense of coherence compared to the group currently only active between 31 to 60 min every day (*p* = 0.039, *M*_*Diff*_ = 3.177, *95% CI*[0.10, 6.26]).

Furthermore, our outcomes pointed out that the majority of cancer patients did sports at an average of 0–2 h every day before their cancer diagnosis and as well as currently. No significant correlations between sports and sense of coherence could be found (Table 2).

When it came to food habits, a mean value of 7.65 out of 12 points was obtained, which stated a moderate healthy nutrition score, but did not significantly correlate with the sense of coherence (Table 2).

### Sense of coherence and CAM usage

In all, the correlations of CAM methods ever and currently used were not in any significant context with the sense of coherence. As many as 208 patients (70.3%) of those who answered the questions on CAM had ever used biological CAM methods, whilst 197 patients (71.4%) who filled out the section about CAM currently used biological CAM methods. No significant correlations between sense of coherence and the seven single biological CAM methods ever used could be noticed (Table 2), whereas a negative correlation between current mistletoe usage and sense of coherence was detected (*r* = − 0.200, *p* = 0.005): the group which currently did not use mistletoe had a greater sense of coherence compared to the group which did. As many as 140 attendants (52.0%) who replied on CAM stated that they had ever used holistic CAM methods, whereas only 72 participants (31.7%) who completed the questionnaire on CAM currently used holistic CAM methods. The correlations of holistic CAM methods ever and currently used were not associated with the sense of coherence (Table 2).

### Sense of coherence and lay aetiology

Of the 28 potential causes for cancer offered in the questionnaire, 11 items presented a significant correlation with sense of coherence (Table 2). Ageing and God’s punishment were positively correlated with the sense of coherence, but without any significance (*p*’s > 0.05).

## Discussion

Our study participants achieved a mean moderate sense of coherence score, which is in accordance with the results of Schumacher et al. (*M* = 47.50), where 2005 people between 18 and 92 years took part in a population representative survey in Germany (Schumacher et al. 2000). Thus, the mean value for the sense of coherence score in cancer patients is within the reference range.

### Sense of coherence and demographic data

Whilst age and sense of coherence do not show any correlation in our population, it is on the contrary reported both to be linked to each other (Eriksson and Lindström 2005; Silverstein and Heap 2015) and to not be connected to each other (Feldt et al. 2003). On the one hand, sense of coherence tends to increase with age (Bredle et al. 2011). We cannot affiliate to this observation, although about one third of our study participants belonged to the group of over 65 years. On the other hand, age does not play any role in the stability of, level of or mean changes in the sense of coherence (Feldt et al. 2003). This leads to the hypothesis that one cannot draw conclusions from age only on the level of sense of coherence as we do not know exactly the actual mental and physical health status of the person at the time interviewed, and, thus, the sense of coherence varies at different stages of age.

Moreover, we cannot find a correlation between sense of coherence and gender amongst our participants. Opposed to our findings, Thomé and Hallberg stated that women with cancer were more vulnerable than their male counterparts in sense of coherence (2004), whilst Gustavsson-Lilius et al. reported no differences in gender in cancer patients, but interestingly stated that female partners of cancer patients represented more distress symptoms than male partners (2007). Thus, we cannot conclude that gender is a predictor for coping abilities and finally for the sense of coherence. Regardless of gender, certain personality traits that are differently pronounced in every person lead to how someone deals or copes with stressors.

In our population, the marital status significantly correlates with sense of coherence on a moderate level. In their cross-sectional study in Japanese young women, Fujitani et al. also describe that the emotional social support from family is positively related to the sense of coherence (2017), while Stefanaki et al. state that widows in Greece present a lower sense of coherence score (2014). We can assume that support from family members has a great impact on a sick person’s wellbeing and, consequently, they should be included in cancer treatment to strengthen their loved-ones.

Interestingly, we cannot find any correlation of religion and sense of coherence, whereas Stefanaki et al. explored a positive association between religion/spiritual beliefs and sense of coherence (2014) and also Zarzycka et al. noted positive relationships between religiosity and sense of coherence in middle-aged men and in female young and late groups (2014). Belonging to a religious community and the belief in God’s guidance might help to comprehend, to cope and to find a meaning in a stressful situation.

In line with our results, Barnard found that high-income employees exhibit a significantly stronger sense of coherence than low-income employees, and that financial wellbeing is positively related to sense of coherence (2016). Thus, healthcare workers should consider that cancer treatment constitutes a financial burden for patients, especially if they do not have enough savings or any health insurance, which partly bears treatment costs. Severe money issues lead to a lowering in sense of coherence and confidence to recover. As a cancer diagnosis already distresses most patients, it is important to check if people are able to manage their financial matters in order for them to worry less about money-related problems and focus on their physical and mental health status. In some cases, involving social workers might be helpful in order to find solutions.

Furthermore, we can ascertain a significant relationship between the sense of coherence and the level of education, showing that the group of patients with a university/college degree scores a greater value in the sense of coherence than the group of patients with a middle school degree. Makara-Studzińska et al. also arrive at this result and observe a significant difference between the respondents with secondary education and undergraduate education (2015). Accordingly, a person who passed through a higher level of education probably had to learn how to deal with certain stressful situations and setbacks and had to find opportunities to handle these situations. This might have helped to develop a stronger sense of coherence over time. Another reason, why well-educated patients have a higher sense of coherence is the capability to comprehend easily and to adapt to stressors. Therefore, physicians should be responsive to the individual patients’ educational background.

In our study, we cannot determine any context between the type of cancer a patient was diagnosed with and the sense of coherence. In contrast, Qiu et al. find an association with the type of primary cancer in patients with brain metastases and sense of coherence (2020). Whereas, a negative correlation between time interval since diagnosis and sense of coherence is seen in our survey, highlighting that a cancer patient has less sense of coherence after a long time has passed. Lindblad et al. come to the opposite conclusion, that the sense of coherence in women with breast cancer is stable over a period of 1 to 3 years after cancer diagnosis (2016). A reason why the sense of coherence is stable in this cohort might be the time factor of only 1 to 3 years compared to a partially longer time factor in our cohort, where about a quarter of our patients had been diagnosed over three or more years ago. Thus, healthcare workers should consider that a long-standing diagnosis, weakens patients and they should be screened to support them to gain confidence and energy to go on.

### Sense of coherence and psychological factors

When it comes to psychological factors in our study, general life satisfaction highly correlates with the sense of coherence, expressing that someone who is satisfied with his or her life in general also has a greater sense of coherence. Moksnes et al. arrive at the same conclusion that sense of coherence is strongly and positively associated with life satisfaction in adolescents (2013). As well, in our outcomes, we see a large association between resilience and sense of coherence, which is in accordance with the results of Festerling et al. and Nygren et al. (2022, 2005). If cancer patients appear depressed and unhappy in general, the team of physicians and psychologists should be consulted. Through a profound conversation, it can be investigated what bothers cancer patients and approaches can be discussed. Patients then feel that they are taken seriously and cared for, and conversely gain satisfaction and resilience, which then helps them to endure and adapt to stressful situations. Similarly, self-efficacy and sense of coherence stand in a great significant context with each other. Trap et al. come to the conclusion that there is a positive and graded correlation between sense of coherence and self-efficacy (2016). Hammond and Niedermann report that patients with chronic diseases who demonstrate high self-efficacy have a better prediction for rehabilitation outcome (2010). Thus, a good communication between healthcare workers and cancer patients is necessary to strengthen the patient and his/her beliefs in himself/herself to be able to cope with hard times and to keep the sense of coherence on a high level.

In addition, spirituality and sense of coherence are linked to each other on a significantly high level in our study. Delgado reported in her survey that high sense of coherence and spirituality were correlated with low stress and high quality of life (2007). Stefanaki et al. also state a strong positive correlation of the sense of coherence score with the spiritual scale (2014). In another study about Iranian mothers with chronically ill children, Avaznejad et al. confirmed that the spiritual wellbeing score has a significant impact on the sense of coherence (2017). Therefore, spiritual beliefs empower patients, which conversely also increases the sense of coherence.

### Sense of coherence and lifestyle

Concerning lifestyle aspects, our data show a small significant correlation between current daily physical activity and sense of coherence, whereas no significant context between daily physical activity before getting diagnosed with cancer and sense of coherence appears. Moyers and Hagger reveal a small non-zero physical activity-sense of coherence correlation with significant heterogeneity in their meta-analysis including 52 studies (2020). Being physically active everyday might strengthen the immune system and, thus, physically fit patients are mentally fitter. Furthermore, there is no significant relationship between the sense of coherence and daily sports before and currently. In contrast, Endo et al. suggest that experience of successive years of sporting activities enhances sense of coherence among college students (2012). Regular sports activities might have a positive influence on mental wellbeing and, thus, patients should be motivated to be active. When looking at dietary habits, we do not find a correlation in sense of coherence and nutrition habits, whereas Lindmark et al. report a low sense of coherence to coincide with a presumably less health-promoting dietary preference (2005). Alike sporting activities, eating healthily should be kept in mind.

### Sense of coherence and CAM usage

When it comes to the CAM methods ever used and currently used, we do not find any significant correlation except for the current mistletoe usage. In literature, mistletoe is described contradictorily and, hence, should be the topic of further investigations. In the study of Hongo et al., no significant difference in the sense of coherence between CAM users and users of both CAM and Western medicine is represented, but they suggest that sense of coherence is related to the selection of CAM (2011). Thus, we can assume that the individual choice to use any CAM method might strengthen the sense of coherence and is a method to actively involve the patient in cancer treatment, but should be further examined, as Hoppe et al. also state (2023).

With respect to mistletoe, the negative correlation provides an important hint to a difference between this integral anthroposophical method and other herbs or supplements. In anthroposophical medicine, diseases as cancer have a meaning which may stem from the patient's former lives. While patients with a good sense of coherence may better cope with a life-threatening disease which does not make sense, those with a low sense of coherence may find the anthroposophical offer of a sense in the disease rather attractive.

### Sense of coherence and lay aetiology

Concerning lay aetiology, we can show that some reasons are significantly linked to the sense of coherence. Welter et al. report that lay aetiology, in general, proves to be influenced by demographics, cancer type, as well as the level of self-efficacy and patient activation (2021), but in context with sense of coherence no data can be found. Especially stress, mental diseases and a lack of sleep seem to negatively influence patients’ ability to cope with stressors and, thus, should be avoided or reduced when undergoing a taxing cancer treatment. Further studies about the sense of coherence and cancer causes could be of interest.

### Limitations

Several limitations of our study need to be mentioned. Although the questionnaire consisted of 10 sub-items, we do not have any information about the current cancer treatment and possible side effects patients’ go through, which might have an influence on their actual mental and physical health status at the time of filling out the questionnaire. We also do not know if participants have other diseases, and therefore maybe need to take further drugs, which might affect cancer treatment, CAM methods used and their general mindset. As data were only collected at one point of time, we do not know if any of the variables changed during cancer treatment and, thus, have an influence on our results. We also have to consider that information is based on subjective statements, which can differ from objective measured information. As patients were categorised into 10 different cancer groups, it was difficult to examine any context of the sense of coherence with the individual cancer types.

Moreover, we do not have any information as to whether patients are actively involved in religious/spiritual communities and/or how important their religious/spiritual belief in general is or how much they are supported by family members/partners and friends. In addition, we also did not consider the fact that data acquisition took place during the COVID pandemic, which portrayed a huge burden for everyone and especially for patients in need of cancer treatment as many hospitals had to postpone or cancel surgeries/cancer treatment due to a shortage of manpower. This might have an impact on several psychological factors collected in our study.

## Conclusion

The sense of coherence has a significant impact on how well someone understands, recognises a sense in, and copes with a cancer disease. Therefore, it is important to integrate the patient and explain the three aspects of coherence. A higher sense of coherence results in higher compliance. Asaba and Okawa described that significant changes in bodily pain in patients receiving cancer chemotherapy showed a buffering effect on the Symptom Distress Scale (SDS) and sense of comprehensibility, sense of manageability, and sense of meaningfulness (2021). Thus, healthcare workers and physicians should consider some factors that can both positively and negatively change the sense of coherence: educational background, financial capacity, support by family members and friends, long-standing diagnosis, general life satisfaction, resilience, self-efficacy, and spirituality. To actively involve the patient in cancer treatment, various CAM methods can be addressed. In addition, it is advisable to motivate the patient to exercise and eat healthily.

## Data Availability

The datasets generated and analysed during this study are available from the corresponding author on appropriate request.

## References

[CR1] Asaba K, Okawa A (2021) Moderating effect of sense of coherence on the relationship between symptom distress and health-related quality of life in patients receiving cancer chemotherapy. Supp Care Cancer. 10.1007/s00520-021-06003-4 10.1007/s00520-021-06003-4 33501621

[CR2] Avaznejad N, Ravanipour M, Motamed N, Bahreini M (2017) Comparative study of the relationship between spiritual well-being and sense of coherence in mothers with chronically iII children in Kerman, Iran, in 2016. Evid Based Care. 7(2):78–83. 10.22038/ebcj.2017.22985.1500

[CR3] Barnard A (2016) Sense of coherence: a distinct perspective on financial well-being. S Afr J Econ Manag Sci. 10.4102/sajems.v19i4.1405

[CR4] Beierlein C, Kemper C, Kovaleva A, Rammstedt B (2013) Kurzskala zur erfassung allgemeiner selbstwirksamkeitserwartungen (ASKU). Method Daten Analy 7(2):28. 10.12758/mda.2013.014

[CR5] Beierlein C, Kovaleva A, László Z, Kemper C, Rammstedt B (2014) Eine Single-Item-Skala zur Erfassung der Allgemeinen Lebenszufriedenheit: Die Kurzskala Lebenszufriedenheit-1 (L-1). GESIS—Leibniz Institut für Sozialwissenschaften. https://www.gesis.org/fileadmin/kurzskalen/working_papers/L1_WorkingPapers_2014-33.pdf. Accessed 05 Mar 2023

[CR6] Bonanno GA, Westphal M, Mancini AD (2011) Resilience to loss and potential trauma. Annu Rev Clin Psychol. 10.1146/annurev-clinpsy-032210-104526 21091190 10.1146/annurev-clinpsy-032210-104526

[CR7] Bredle JM, Salsman JM, Debb SM, Arnold BJ, Cella D (2011) Spiritual well-being as a component of health-related quality of life: the functional assessment of chronic illness therapy-spiritual well-being scale (FACIT-Sp). Religions. 10.3390/rel2010077

[CR8] Ciarlo G, Ahmadi E, Welter S, Hübner J (2021) Factors influencing the usage of complementary and alternative medicine by patients with cancer. Complement Ther Clin Pract. 10.1016/j.ctcp.2021.101389 33932731 10.1016/j.ctcp.2021.101389

[CR9] Delgado C (2007) Sense of coherence, spirituality, stress and quality of life in chronic illness. J Nurs Scholarsh. 10.1111/j.1547-5069.2007.00173.x 17760795 10.1111/j.1547-5069.2007.00173.x

[CR10] Duggal D, Sacks-Zimmerman A, Liberta T (2016) The impact of hope and resilience on multiple factors in neurosurgical patients. Cureus. 10.7759/cureus.849 27909637 10.7759/cureus.849 PMC5120968

[CR11] Endo S, Kanou H, Oishi K (2012) Sports activities and sense of coherence (SOC) among college students. Int J Sport Health Sci. 10.5432/ijshs.201114

[CR12] Eriksson M, Lindström B (2005) Validity of Antonovsky’s sense of coherence scale: a systematic review. J Epidemiol Commun Health. 10.1136/jech.2003.018085 10.1136/jech.2003.018085 PMC1757043 15911640

[CR13] Feldt T, Leskinen E, Kinnunen U, Ruoppila I (2003) The stability of sense of coherence: comparing two age groups in a 5-year follow-up study. Person Individ Differ. 10.1016/S0191-8869(02)00325-2

[CR14] Festerling L, Buentzel J, Fischer von Weikersthal L, Junghans C, Zomorodbakhsch B, Stoll C, Prott FJ, Fuxius S, Micke O, Richter A, Sallmann D, Huebner J, Hoppe C (2022) Resilience in cancer patients and how it correlates with demographics, psychological factors, and lifestyle. J Cancer Res Clin Oncol. 10.1007/s00432-022-04480-6 36396875 10.1007/s00432-022-04480-6 PMC10349752

[CR15] Fujitani T, Ohara K, Kouda K, Mase T, Miyawaki C, Momoi K, Okita Y, Furutani M, Nakamura H (2017) Association of social support with gratitude and sense of coherence in Japanese young women: a cross-sectional study. Psychol Res Behav Manag. 10.2147/PRBM.S137374 28721109 10.2147/PRBM.S137374 PMC5499777

[CR16] Gustavsson-Lilius M, Julkunen J, Keskivaara P, Hietanen P (2007) Sense of coherence and distress in cancer patients and their partners. Psychooncology. 10.1002/pon.1173 17323314 10.1002/pon.1173

[CR17] Hammond A, Niedermann K (2010) Chapter 6 - Patient education and self-management. In: Dziedzic K, Hammond A (eds) Rheumatology: evidence-based practice for physiotherapists and occupational therapists, 1st edn. Churchill Livingstone, Edinburgh, pp 77–97

[CR18] Höh JC, Schmidt T, Hübner J (2018) Physical activity among cancer survivors-what is their perception and experience? Supp Care Cancer. 10.1007/s00520-017-3977-0 10.1007/s00520-017-3977-0 29168034

[CR19] Hongo A, Takahashi Y, Soejima K, Tanaka A, Mamoru ITO (2011) Selection of complementary and alternative medicine and a sense of coherence. J Showa Med Assoc 71(4):398–407

[CR20] Hoppe C, Buntzel J, Von Weikersthal LF, Junghans C, Zomorodbakhsch B, Stoll C, Prott FJ, Fuxius S, Micke O, Richter A, Sallmann D, Hubner J (2023) Usage of complementary and alternative methods, lifestyle, and psychological variables in cancer care. In Vivo 37(1):106–114 36593056 10.21873/invivo.13059 PMC9843784

[CR21] Huebner J, Micke O, Muecke R, Buentzel J, Prott FJ, Kleeberg U, Senf B, Muenstedt K (2014) User rate of complementary and alternative medicine (CAM) of patients visiting a counseling facility for CAM of a German comprehensive cancer center. Anticancer Res 34(2):943–948 24511037

[CR22] Huey CWT, Palaganas JC (2020) What are the factors affecting resilience in health professionals? A synthesis of systematic reviews. Med Teach. 10.1080/0142159X.2020.1714020 31984844 10.1080/0142159X.2020.1714020

[CR23] Johnson F, Wardle J, Griffith J (2002) The adolescent food habits checklist: reliability and validity of a measure of healthy eating behaviour in adolescents. Eur J Clin Nutr. 10.1038/sj.ejcn.1601371 12080404 10.1038/sj.ejcn.1601371

[CR24] Josfeld L, Krüger L, Büntzel J, Zomorodbakhsch B, Hübner J (2022) Self-efficacy in relation to the use of complementary and alternative medicine, lifestyle choices and cancer aetiology. J Cancer Res Clin Oncol. 10.1007/s00432-021-03857-3 34812932 10.1007/s00432-021-03857-3 PMC9470719

[CR25] Konaszewski K, Kolemba M, Niesiobędzka M (2021) Resilience, sense of coherence and self-efficacy as predictors of stress coping style among university students. Curr Psychol. 10.1007/s12144-019-00363-133935472

[CR26] Leitlinienprogramm Onkologie (Deutsche Krebsgesellschaft DK, AWMF) (2021) S3-Leitlinie Komplementärmedizin in der Behandlung von onkologischen PatientInnen. Langversion 1.1. AWMF Registernummer: 032/055OL. https://www.leitlinienprogramm-onkologie.de/leitlinien/komplementaermedizin/. Accessed 05 Mar 2023

[CR27] Leppert K, Koch B, Brähler E, Strauß B (2008) Die Resilienzskala (RS)—Überprüfung der Langform RS-25 und einer Kurzform RS-13. Klin Diagn Eval 1(2):226–243

[CR28] Lindblad C, Sandelin K, Petersson LM, Rohani C, Langius-Eklöf A (2016) Stability of the 13-item sense of coherence (SOC) scale: a longitudinal prospective study in women treated for breast cancer. Qual Life Res. 10.1007/s11136-015-1114-4 26319753 10.1007/s11136-015-1114-4

[CR29] Lindmark U, Stegmayr B, Nilsson B, Lindahl B, Johansson I (2005) Food selection associated with sense of coherence in adults. Nutr J. 10.1186/1475-2891-4-9 15737236 10.1186/1475-2891-4-9 PMC554973

[CR30] Makara-Studzińska M, Sidor K, Kalinowski P (2015) Analysis of the impact of education level on the sense of coherence and opinion concerning carrying out preventive vaccination. J Pre Clin Clin Res. 10.5604/18982395.1157575

[CR31] Manomenidis G, Panagopoulou E, Montgomery A (2019) Resilience in nursing: the role of internal and external factors. J Nurs Manag. 10.1111/jonm.12662 30066352 10.1111/jonm.12662

[CR32] Moksnes UK, Løhre A, Espnes GA (2013) The association between sense of coherence and life satisfaction in adolescents. Qual Life Res. 10.1007/s11136-012-0249-9 22886139 10.1007/s11136-012-0249-9

[CR33] Moyers S, Hagger M (2020) Physical activity and sense of coherence: a meta-analysis. Int Rev Sport Exercise Psychol. 10.1080/1750984X.2020.1846068

[CR34] Nygren B, Aléx L, Jonsén E, Gustafson Y, Norberg A, Lundman B (2005) Resilience, sense of coherence, purpose in life and self-transcendence in relation to perceived physical and mental health among the oldest old. Aging Ment Health. 10.1080/1360500114415 16019292 10.1080/1360500114415

[CR35] Omiya T, Deguchi N, Togari T, Yamazaki Y (2020) Factors influencing sense of coherence: family relationships high school life and autism spectrum tendency. Children (basel). 10.3390/children7090108 32825759 10.3390/children7090108 PMC7552669

[CR36] Qiu X, Zhang N, Pan SJ, Zhao P, Wu BW (2020) Sense of coherence and health-related quality of life in patients with brain metastases. Front Psychol. 10.3389/fpsyg.2020.01516 32714254 10.3389/fpsyg.2020.01516 PMC7343871

[CR37] Schumacher J, Leppert K, Gunzelmann T, Strauß B, Brähler E (2005) Die Resilienzskala – Ein Fragebogen zur Erfassung der psychischen Widerstandsfähigkeit als Personmerkmal. Z Klin Psychol Psychiatr Psychother 53(1):16–39

[CR38] Schumacher J, Wilz G, Gunzelmann T, Brähler E (2000) Die Sense of Coherence Scale von Antonovsky Teststatistische Überprüfung in einer repräsentativen Bevölkerungsstichprobe und Konstruktion einer Kurzskala. Psychother Psychosom Med Psychol. 10.1055/s-2000-9207 11199111 10.1055/s-2000-9207

[CR39] Silverstein M, Heap J (2015) Sense of coherence changes with aging over the second half of life. Adv Life Course Res. 10.1016/j.alcr.2014.12.002 26047844 10.1016/j.alcr.2014.12.002

[CR40] Stefanaki IN, Shea S, Linardakis M, Symvoulakis EK, Wynyard R, Lionis C (2014) Exploring the association of sense of coherence, and spiritual and religious beliefs in a rural population group on the island of Crete Greece. Int J Psychiatry Med. 10.2190/PM.47.3.c 25084818 10.2190/PM.47.3.c

[CR41] Sung H, Ferlay J, Siegel RL, Laversanne M, Soerjomataram I, Jemal A, Bray F (2021) Global cancer statistics 2020: GLOBOCAN estimates of incidence and mortality worldwide for 36 cancers in 185 Countries. CA Cancer J Clin. 10.3322/caac.21660 33538338 10.3322/caac.21660

[CR42] Thomé B, Hallberg IR (2004) Quality of life in older people with cancer – a gender perspective. Eur J Cancer Care (engl). 10.1111/j.1365-2354.2004.00471.x 15606713 10.1111/j.1365-2354.2004.00471.x

[CR43] Trap R, Rejkjær L, Hansen EH (2016) Empirical relations between sense of coherence and self-efficacy National Danish Survey. Health Promot Int. 10.1093/heapro/dav052 26069296 10.1093/heapro/dav052

[CR44] Welter S, Keinki C, Ahmadi E, Huebner J (2021) Lay etiology, self-efficacy and patient activation among cancer patients. Cancer Invest. 10.1080/07357907.2021.1878528 33475001 10.1080/07357907.2021.1878528

[CR45] Zarzycka B, Rydz E (2014) Centrality of religiosity and sense of coherence: a cross-sectional study with polish young, middle and late adults. Int J Soc Sci Stud. 10.11114/ijsss.v2i2.346

